# Transient Receptor Potential Cation Channel Subfamily V Member 4 Mediates Pyroptosis in Chronic Obstructive Pulmonary Disease

**DOI:** 10.3389/fphys.2021.783891

**Published:** 2021-12-24

**Authors:** Yafei Rao, Xiaoyan Gai, Jing Xiong, Yanqing Le, Yongchang Sun

**Affiliations:** Department of Respiratory and Critical Care Medicine, Peking University Third Hospital, Beijing, China

**Keywords:** chronic obstructive pulmonary disease (COPD), cigarette smoke, TRPV4, pyroptosis, inflammation, mitochondrial dysfunction

## Abstract

TRPV4, a calcium permeable cation selective channel, was found to be involved in chronic obstructive pulmonary disease (COPD) through releasing ATP and IL-1β. Pyroptosis, a newly discovered pro-inflammatory cell death, was induced by cigarette smoke (CS) in airway epithelial cells (AECs). More recent studies indicated that blocking Ca^2+^ influx effectively inhibited pyroptosis. Therefore, we asked whether TRPV4 mediated CS-induced pyroptosis of AECs and hence participated in the pathogenesis of COPD. We found that pyroptosis and TRPV4 were upregulated in AECs from patients with COPD and long-term CS-exposed mice. Moreover, pharmacological inhibition or knockdown of TRPV4 function alleviated CS extract (CSE)-induced pyroptosis by inhibiting NACHT, LRP, PYD domains-containing protein 3 (NLRP3) inflammasome/activated caspase-1/gasdermin D pathway, decreasing the number of PI positive cells and lactate dehydrogenase (LDH) release, decreasing the expression of pro- inflammatory interleukin gene (IL)-1β, IL-8, and IL-18 expression, as well as increasing anti-inflammatory gene expression [NAD(P)H quinone dehydrogenase 1 (*NQO1)*, superoxide dismutase 2 (mitochondrial) (*MNSOD)*, and catalase, (*CAT)*]. Moreover, pharmacological inhibition or knockdown of TRPV4 function significantly relieved CSE-induced mitochondrial damage including decreased mitochondrial membrane potential, mitochondrial fusion protein (OPA1, MFN2) expression, and increased mitochondrial fission protein (DRP1, MFF) expression. Taken together, these findings indicate that TRPV4 mediates AEC pyroptosis via NLRP3/caspase-1/GSDMD pathway in COPD.

## Introduction

Chronic obstructive pulmonary disease (COPD) is featured by chronic airway inflammation and pulmonary parenchymal destruction (Singh et al., 2019). Airway epithelial cells (AECs) form the first barrier of the respiratory system against irritants from the environment and play a critical role in the development or progression of COPD (Brusselle et al., 2011; Tuder and Petrache, 2012; Barnes, 2013).

Pyroptosis, a form of lytic cell death resulting from pathogenic infection or endogenous challenge, drives inflammation through inflammatory cytokines and danger molecules (Jorgensen and Miao, 2015; Broz and Dixit, 2016; Rathinam et al., 2019). Pyroptosis has been shown to be increased in a variety of diseases, such as sepsis, acute pancreatitis, renal ischemia reperfusion injury, diabetic cardiomyopathy and Alzheimer’s disease (Man et al., 2017; Manthiram et al., 2017; Swanson et al., 2019; Feng et al., 2020; Xiao et al., 2020; Xie et al., 2020; Gao et al., 2021). Emerging evidence indicates that cigarette smoke (CS) exposure can induce pyroptosis of AECs, suggesting a critical role of pyroptosis in the pathogenesis of COPD (Zhang et al., 2021).

Transient receptor potential cation channel subfamily V member 4 (TRPV4) belongs to the transient receptor potential (TRP) family, comprising of calcium permeable cation selective channels (Clapham, 2003; Negri et al., 2019). Accumulating evidence indicates that TRPV4 is involved in a variety of lung diseases, including cough (Bonvini et al., 2016; Bonvini and Belvisi, 2017), asthma (Yao et al., 2019), COPD (Baxter et al., 2014), idiopathic pulmonary fibrosis (IPF) (Riteau et al., 2010), and acute respiratory distress syndrome (Balakrishna et al., 2014). Interestingly, a previous study suggested that TRPV4 participated in the pathogenesis of COPD, by observing that TRPV4 mRNA was upregulated in lung tissues, alveolar macrophages and bronchial epithelial cells from COPD patients. Additionally, cigarette smoke extract (CSE)-induced ATP and IL-1β release was mediated by TRPV4 (Baxter et al., 2014). But the exact role of TRPV4 in the pathogenesis of COPD still awaits investigation. More recently, another study indicated that blocking Ca^2+^ influx effectively inhibited pyroptosis (Wang et al., 2020). Therefore, we asked whether TRPV4 played a role in the pathogenesis of COPD by mediating pyroptosis of AECs.

In this study, we evaluated pyroptosis and TRPV4 expression in AECs from patients with COPD and long-term CS-exposed mice. We found that pyroptosis and TRPV4 were significantly upregulated in AECs from COPD patients and long-term CS-exposed mice. Moreover, pharmacological inhibition or knockdown of TRPV4 function attenuated protein expressions of NLRP3 inflammasome, activated caspase-1 and gasdermin D-N (GSDMD-N), decreased the number of PI positive cells and lactate dehydrogenase (LDH) release, decreased the expressions of pro- inflammatory interleukin (IL)-1β, IL-8, and IL-18, while increased anti-inflammatory gene expressions including [NAD(P)H quinone dehydrogenase 1 (*NQO1)*, superoxide dismutase 2 (mitochondrial) (*MNSOD)* and catalase (*CAT)*]. Furthermore, pharmacological inhibition or knockdown of TRPV4 function significantly relieved CSE-induced mitochondrial damage, as manifested by increased mitochondrial membrane potential, mitochondrial fusion protein (OPA1, MFN2) expression and decreased mitochondrial fission protein (DRP1, MFF) expression.

## Materials and Methods

### Human Subjects

The human study was approved by the Ethics Review Committee of Peking University Third Hospital. All study subjects signed an informed consent form. Totally, 24 subjects were recruited and subdivided into a healthy non-smoking (HNS) group, a smoker group and a COPD group. HNS controls were defined as never-smokers having a post-bronchodilator FEV_1_/FVC ≥ 0.7. Smokers were defined as having a smoking history of ≥ 10 pack-years with a post-bronchodilator FEV_1_/FVC ≥ 0.7. COPD patients were defined as having ≥ 10 pack-years of smoking history, and a post-bronchodilator FEV_1_/ FVC < 0.7. All COPD patients recruited were clinically stable. The study subjects had received lung surgery for solitary tumors, and lung tissues with a maximum distance from the tumor were collected by a pathologist.

### The Mouse Model of Chronic Obstructive Pulmonary Disease

All the procedures and protocols carried out in the mouse experiments were approved by Animal Care Committee of Peking University Third Hospital. 6–8week old C57BL/6 female mice, weight 22–25 g, were provided by Beijing Vital River Experimental Animal Company (Beijing, China). All the mice were housed in a specific pathogen-free facility with free access to sterilized food and water. These mice were exposed to filtered air or CS (Baisha cigarettes with filter, Hunan, China) with a nose−only smoke exposure system (SG−300; SIBATA, Saitama, Japan), which has been described in our previous study (Zhou et al., 2019; Xiong et al., 2020). Each mouse was exposed to cigarettes or filtered air for two times a day, 50 min each time with 20-min smoke -free intervals, 5 days a week, for 24 weeks consecutively.

### Lung Histology and Measurement of Emphysema

Mouse lung tissues were obtained, fixed with 4% paraformaldehyde for 24 h, embedded with paraffin, then cut into sections of 4 μm thickness, followed by H&E staining. The airspace enlargement in mice was quantified by the mean linear intercept (MLI) and destruction of alveolar walls in mice was quantified by destructive index (DI), which has been described in our previous studies (Zhou et al., 2019; Xiong et al., 2020).

### Immunohistochemistry of Lung Tissues

Lung tissues were cut into 5 μm sections, incubated in 0.3% H_2_O_2_-CH_3_OH for 15 min for blocking endogenous peroxidase activity, then treated with citrate buffer (pH 6.0) using a microwave oven for 15 min to retrieve antigen, and followed by blocking with 5% bovine serum albumin for 30 min at room temperature (RT). Subsequently, the tissues were incubated overnight with antibodies against TRPV4 (1:100, Alomone labs, Jerusalem, Israel), human gasdermin D (GSDMD) (1:200, Abcam, Cambridge, United Kingdom), mouse GSDMD (1:1,500, Bioss Biotechnology Co., Ltd, Beijing, China), human gasdermin D N-terminal fragment (GSDMD-N) (1:400, Abcam), and mouse gasdermin D C-terminal fragment (GSDMD-C) (1:400, Abcam). The tissues were then incubated with horseradish peroxidase (HRP)-conjugated goat anti-rabbit IgG (ZSGB-Bio, Beijing, China) at 37°C for 30 min. Finally, slides were visualized by staining with a 3,3′-Diaminobenzidine (DAB) detection system kit (ZSGB-Bio). Images were photographed by a microscope and analyzed by Image-Pro Plus 6.0 software (Media Cybernetics, MD, United States).

### Cell Culture and Antagonist Treatment

Human bronchial epithelial cells (16HBEs) were purchased from Bai Ye Biotechnology Center (Shanghai, China). Cells were cultured in Roswell Park Memorial Institute medium supplemented with 10% fetal calf serum (Gibco, Grand Island, NY, United States), 1% penicillin/streptomycin (Gibco, Grand Island, NY, United States) at 37°C with 5% CO_2_. 16HBEs were pretreated with 10 μM of a TRPV4 antagonist [GSK205 (catalog number HY120691A, MCE, Monmouth Junction, NJ, United States)] for 1 h and then stimulated with medium only or with CSE for another 24 h.

### Short Interfering RNA Knockdown of Transient Receptor Potential Cation Channel Subfamily V Member 4

For transient transfection, cells were cultured in six-well culture plates overnight prior to transfection. Short interfering (si) RNA or control oligonucleotide (GenePharma, Jiang Su, China) were incubated with 4 μl of JetPRIME^®^ in 200 μl of jetPRIME buffer (Polyplus-transfection) for 15 min at room temperature. A final concentration of 50 nM of TRPV4 siRNA or control oligonucleotide were added to cell culture plates. After 24 h, CSE was added and stimulated for another 24 h. Finally, cells were harvested for subsequent experiments.

### Cigarette Smoke Extract Preparation

CSE was prepared according to our previous study (Zhou et al., 2019). Briefly, five cigarettes (Baisha, China Tobacco Industry Co., Ltd., Hunan, China) were bubbled through 10 ml medium at a constant velocity (Hyclone, Logan, UT, United States). The solution was filtered through a 0.22 μm filter, which was served as the 100% CSE work solution.

### Cell Viability Assessment

Cell viability was detected by the cell counting Kit-8 (CCK8) assay kit (KeyGEN Biotechnology Co., Ltd., Jiangsu, China). In brief, Cells were treated with medium or different concentrations of CSE or GSK205 for 24 h, then washed with PBS for three times and incubated with CCK8 reagent for another 2 h. Subsequently, the absorbance was assessed by spectrophotometer at 450 nm.

### Western Blotting Analysis

To assess the protein expression levels of TRPV4, NLRP3, Caspase-1, GSDMD, GSDMD-N, GSDMD-C, dynamin-related protein 1 (DRP1), mitochondrial fission factor (MFF), OPA1 mitochondrial dynamin like GTPase (OPA1), and mitofusin 2 (MFN2), lung tissue or 16HBE cells were lysed and equal amount of protein was subjected to 8 or 10% SDS-PAGE, subsequently, protein was transferred to 0.22 μm PVDF membranes (Merck-Millipore, Carrigtwohill, Ireland). The membranes were blocked with 5% non-fat milk powder and then incubated with antibodies overnight at 4°C: GAPDH, β-actin, DRP1, MFF, OPA1, MFN2 (1:1,000, Cell Signaling Technology, Danvers, MA, United States), Caspase-1, NLRP3 (1:1,000, RD system, Minneapolis, MN, United States), GSDMD, GSDMD-N, and GSDMD-C (1:1,000, Abcam, Cambridge, United Kingdom).

Membranes were then washed and incubated with HRP-conjugated donkey anti-mouse IgG or antirabbit IgG antibody for 1 h at RT. Finally, membranes were visualized with enhanced chemiluminescence (MerckMillipore, Carrigtwohill, Ireland). Quantitative images were analyzed using Image J software.

### Quantitative Real-Time Reverse Transcription PCR

Gene expressions of TRPV4, *IL1B*, *IL8*, *IL18*, *NQO1* [NAD(P)H quinone dehydrogenase 1], *MNSOD* [superoxide dismutase 2 (mitochondrial), and *CAT* (catalase)] were determined by qRT-PCR. RNA was isolated by TRIzol reagent (Thermo Fisher Scientific, MA, United States) and the concentration was determined by Nano Drop 2000. Then RNA was reverse transcribed into cDNA using HiScript III RT Supermix for qPCR(+gDNA wiper) Kit (Vazyme, Nanjing, China). The qPCR reactions were performed on the Applied Biosystems^®^ QuantStudio^®^ 5 in a 20 μl reaction system by using ChamQ Universal SYBR qPCR Master Mix Kit (Vazyme, Nanjing, China). The primer sequences were: human *TRPV4*: 5′-GGCTTTTCCTCTCTCCTCCC-3′ (forward) and 5′-AGGGTGGACTCCAGCAGAT-3′ (reverse); mouse *TRPV4*: 5′-TCCTGAGGCCGAGAAGTACA-3′ (forward) and 5′-TCCC CCTCAAACAGATTGGC-3′ (reverse); human *GAPDH*/*Gapdh*: 5′-AAATCAAGTGGGGCGATGCTG-3′ (forward) and 5′-GCA GAGATGATGACCCTTTTG-3′(reverse); mouse *GAPDH*/ *Gapdh*: 5′-AAATGGTGAAGGTCGGTGTGAAC-3′ (forward) and 5′-CAACAATCTCCACTTTGCCACTG-3′(reverse); *IL-1*β: 5′-ACTGAGAGTGATTGAGAGTGGAC-3′ (forward) and 5′-AACCCTCTGCACCCAGTTTTC-3′ (reverse); *IL-*8: 5′-AT GATGGCTTATTACAGTGGCAA-3′ (forward) 5′-GTCGGAGA TTCGTAGCTGGA-3′ (reverse); *IL-18*: 5′-TCTTCATTGACC AAGGAAATCGG-3′ (forward) and 5′-TCCGGGGTGCATTAT CTCTAC-3′ (reverse); *NQO1*: 5′-CAGTGGCATGCACCCA GGGAA-3′ (forward) and 5′-GCATGCCCCTTTTAGCCTTG GCA-3′ (reverse); *MNSOD*: 5′-ACAGGCCTTATTCCACTGCT-3′ (forward) and 5′-CAGCATAACGATCGTGGTTT-3′ (reverse); and *CAT*: 5′-TAAGACTGACCAGGGCATC-3′ (forward) and 5′-CAAACCTTGGTGAGATCGAA-3′ (reverse). The real-time PCR conditions were: 95°C for 15 min, followed by 40 cycles of 95°C for 10 s and 60°C for 32 s. Relative gene expression to control was calculated with the 2^–Δ^ ^Δ^ *^CT^* method. Gene expression was calculated relative to that of *GAPDH/Gapd*h in triplicates.

### Ca^2+^ Influx Measurement

Calbryte 520 AM (AAT Bioquest, San Francisco, CA, United States) was used to assess the intracellular Ca^2+^ levels. In brief, cells were loaded with 2 mM Calbryte 520 AM at 37°C in the dark for 1 h, and then washed with PBS. Thereafter, cells were stimulated with CSE for 20 min. The intracellular Ca^2+^ influx was recorded by a confocal laser scanning microscope for 20 consecutive minutes, at 5 s per frame.

### Measurement of Intracellular Reactive Oxygen Species

2′,7′-dichlorofluorescin diacetate (DCFH-DA) (Beyotime Biotechnology Co., Ltd., Shanghai, China) was used to assess the intracellular ROS with fluorescent microscopy. In brief, cells were pretreated with GSK205 10 μM for 1 h, and then stimulated with medium only or CSE for another 24 h. These cells then underwent incubation with 10 μM DCFH-DA for 20 min at 37°C. Finally, the intracellular ROS was assessed at 488/525 nm using fluorescent microscopy (Leica, Germany) and analyzed by Image-Pro Plus 6.0 software (Media Cybernetics, MD, United States).

### Measurement of Mitochondrial Reactive Oxygen Species

Mitochondrial ROS was assessed by MioSox Red (Invitrogen, Life Technologies, Carlsbad, CA, United States). Briefly, after corresponding treatment, cells were stained with 5 μM Mio Sox Red in the dark for 10 min. The mitochondrial ROS was determined at 510/580 nm using fluorescent microscopy (Leica, Germany) and analyzed by Image-Pro Plus 6.0 software (Media Cybernetics, MD, United States).

### Lactate Dehydrogenase Assay

LDH was assessed using an LDH assay kit (KeyGEN Biotechnology Co., Ltd). Briefly, cells were handled as described above, then the supernatants were harvested and assessed for LDH release with a microplate reader at 490 nm according to the manufacturer’s instruction.

### Hoechst 33342/PI Staining

Hoechst 33342/PI staining was measured using apoptosis and necrosis Assay Kit (Beyotime Biotechnology Co., Ltd., Shanghai, China). After corresponding treatments described above, cells were stained with 5 μl Hoechst 33342 and 5 μl PI in 1 ml staining buffer solution on ice in the dark for 20 min. Then, PI positive cells were photographed by confocal microscopy.

### Measurement of Mitochondrial Membrane Potential

MMP was assessed with Mitotracker deep red (Invitrogen, Life Technologies). After corresponding treatment, cells were incubated with 50 nM Mitotracker deep red for 30 min at 37°C, and then detected at 644/655 nm by flow cytometry.

### Mitochondrial Morphology Detection

After corresponding treatment, mitochondria were labeled with the MitoTracker Green (Beyotime Biotechnology Co., Ltd., Shanghai, China) for 30 min in the dark. The mitochondrial morphology was photographed by a confocal microscope (Leica SP5, Germany). MitoTracker Green fluorescence intensity, mitochondrial Aspect Ratio and Form Factor was analyzed by Image J software. A minimum of 20 mitochondria were analyzed.

After corresponding treatment, cells were collected and washed with PBS for subsequent transmission electron microscopic (TEM) detection. Firstly, cells were added with Glutaraldehyde (2.5%) carefully and fixed at 4°C for 1.5 h. Then, the mixture was washed three times with sucrose buffer, fixed, dehydrated, soaked, and embedded, ultrathin sectioned and heavy metal stained. The mitochondrial morphology was assessed by JEM1400 PLUS. Mitochondrial Aspect Ratio and Form Factor in mitochondrial were calculated using Image J software. A minimum of 30 mitochondria were analyzed.

### Statistical Analysis

All data were shown as mean ± standard deviation (SD). Statistical analysis was processed with GraphPad Prism 7. Students’ *t*-tests or one-way ANOVA with Bonferroni *post hoc* test (equal variances assumed) or Dunnett’s T3 *post hoc* test (equal variances not assumed) were used when applicable. *P*-values less than 0.05 were considered statistically significant.

## Results

### Clinical Characteristics of Human Subjects

Eight COPD patients, 8 smokers, and 8 HNS controls were enrolled. Table 1 shows the clinical data of the subjects.

**TABLE 1:** Clinical characteristics of the study subjects.

	HNS	Smoker	COPD
Subjects (all male)	8	8	8
Age (years)	61.43 ± 15.96	64.75 ± 7.96	68.13 ± 8.45
FEV1/FVC (%), post-bronchodilator	79.14 ± 3.76	76.09 ± 4.09	63.82 ± 3.8*
FEV%pred, post-bronchodilator	98.86 ± 22.45	86.16 ± 13.16	65.61 ± 11.35*
Smoking index (pack-years)	0	41.38 ± 23.01^#^	55 ± 21.38*

*Smoking index = average number of cigarettes per day (pack) × number of years of smoking history (years); FEV1, forced expiratory volume in 1 s; FVC, force vital capacity; HNS, healthy none smoker; COPD, chronic obstructive pulmonary disease.*

*^#^There was significant difference between Smokers and the HNS.*

*There was significant difference between Smokers and COPD patients.

### Increased Pyroptosis of Airway Epithelial Cells in Human and Mouse Chronic Obstructive Pulmonary Disease

**FIGURE 1:**
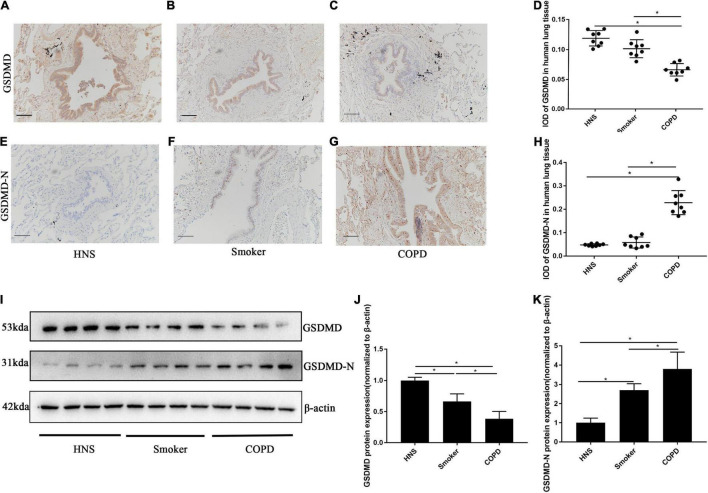
GSDMD-N expression was upregulated in AECs from patients with COPD. **(A–C)** Representative immunohistochemical staining of GSDMD in AECs of HNS, Smoker and COPD groups. **(E–G)** Representative immunohistochemical staining of GSDMD-N in AECs of HNS, Smoker and patients with COPD. **(D–H)** The integrated optical density (IOD) in immunohistochemistry of GSDMD and GSDMD-N. Bar: 100 μm. *N* = 8 per group. *P < 0.05. **(I–K)** GSDMD, GSDMD-N protein expression in lung tissue homogenates from HNS, Smoker and patients with COPD were detected by Western blotting. *N* = 8 per group. *P < 0.05.

A previous *in vitro* study has implicated pyroptosis in the pathogenesis of COPD (Zhang et al., 2021), however, the expression of pyroptosis in human and mouse COPD were still unknown. Therefore, we investigated the status of pyroptosis of AECs in patients with COPD, smokers and HNS, by performing immunohistochemical staining of lung sections. Patients with COPD showed reduced protein level of GSDMD (Figures 1A–D), but increased protein level of GSDMD-N (Figures 1E–H) in AECs, as compared to smokers and HNS. Moreover, Western blotting analysis revealed decreased GSDMD but increased GSDMD-N protein levels in lung tissue homogenates from COPD patients as compared to smokers and HNS (Figures 1I–K), indicating enhanced pyroptosis in patients with COPD.

**FIGURE 2:**
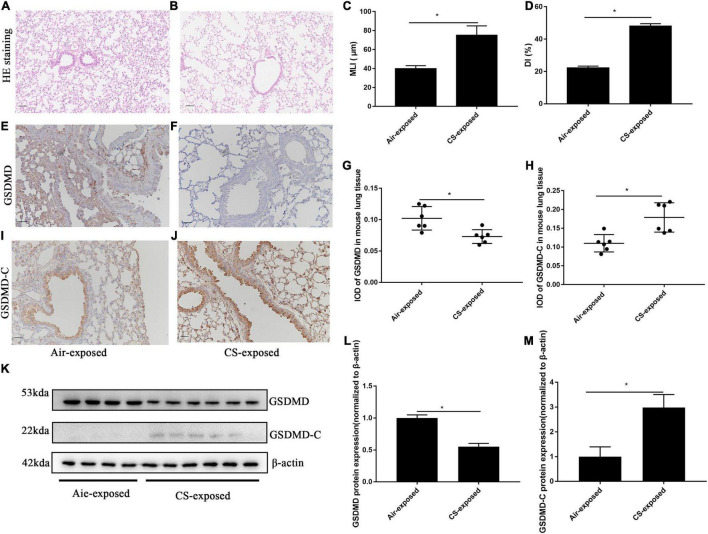
GSDMD-C expression was upregulated in AECs from mouse COPD. **(A,B)** H&E-stained lung sections Bar: 50 μm. **(C,D)** Mean linear intercept (MLI) and destructive index (DI) was measured. **(E,F)** Representative immunohistochemical staining of GSDMD in AECs of CS–exposed group and air–exposed group. **(I,J)** Representative immunohistochemical staining of GSDMD-C in AECs of air–exposed and CS–exposed mice. **(G,H)** The integrated optical density (IOD) in immunohistochemistry of GSDMD and GSDMD-C. Bar: 50 μm. *N* = 6 per group. *P < 0.05. **(K–M)** GSDMD, GSDMD-C protein expression in lung tissue homogenates from air-exposed mice (*N* = 4) and CS-exposed mice (*N* = 6) were detected by Western blotting. *P < 0.05.

We also performed immunohistochemistry for the detection of pyroptosis of AECs in lung sections from mice with CS-induced COPD. Mice in the 6-month CS-exposed group showed enlargement of airway spaces (Figures 2A,B), with significant increase in MLI and DI (Figures 2C,D), which is in line with typical changes of COPD. Consistent with the observations in human subjects, CS-exposed mice showed reduced protein level of GSDMD (Figures 2E–G), but increased protein level of GSDMD-C (a pyroptosis marker in mice) in AECs, as compared to air-exposed mice (Figures 2H–J). Control antibody staining was illustrated in Supplementary Figures 1, 2. Western blotting analysis also demonstrated reduced GSDMD but increased GSDMD-C protein levels in lung tissue homogenates from CS-exposed mice as compared to air-exposed mice (Figures 2K–M). Collectively, these data demonstrated that there was a significant increase in pyroptosis of AECs in human and mouse COPD.

### Transient Receptor Potential Cation Channel Subfamily V Member 4 Expression Was Upregulated in Airway Epithelial Cells From Human and Mouse Chronic Obstructive Pulmonary Disease

**FIGURE 3:**
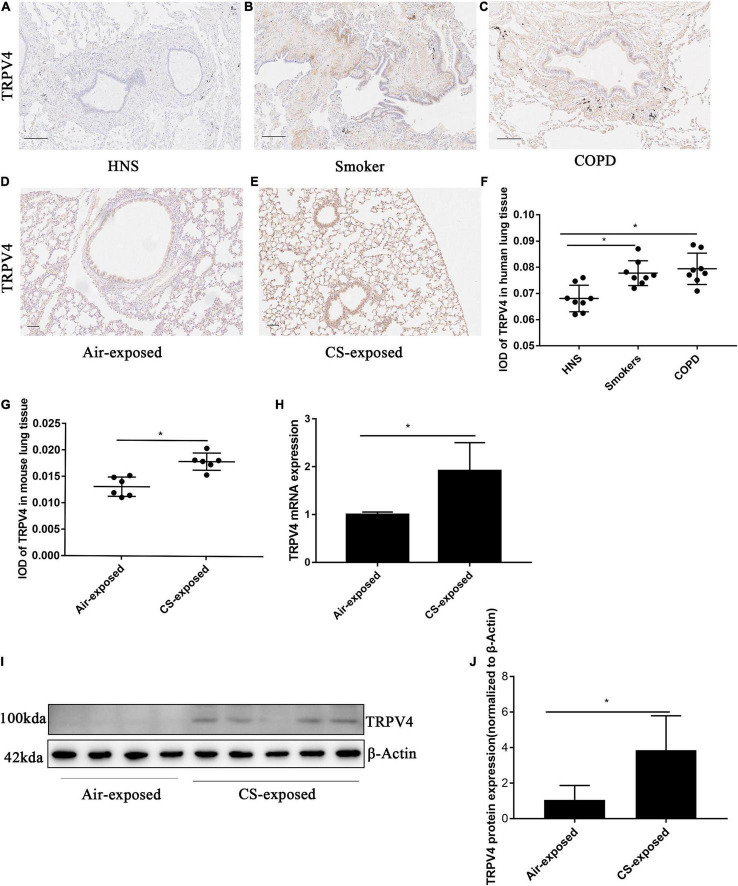
TRPV4 expression was up-regulated in AECs in human and mouse COPD. **(A–C)** Representative immunohistochemical staining of TRPV4 in lung sections of HNS, Smoker and patients with COPD. **(F)** The integrated optical density (IOD) in immunohistochemistry of TRPV4. Bar: 100 μm. *N* = 8 per group. *P < 0.05. **(D,E)** Representative immunohistochemical staining of TRPV4 in AECs of air–exposed mice and CS–exposed mice. **(G)** The integrated optical density (IOD) in immunohistochemistry of TRPV4. Bar: 50 μm. *N* = 6 per group. *P < 0.05. **(H)** The lung homogenates of CS–exposed mice displayed increased *TRPV4* mRNA expression compared with air–exposed mice. **(I,J)** The lung homogenates of CS–exposed mice (*N* = 5) demonstrated increased TRPV4 expression at protein level compared with air–exposed mice (*N* = 4). *P < 0.05.

A previous study observed increased *TRPV4* mRNA in lung homogenates from patients with COPD in relative to HNS and smokers (Baxter et al., 2014). To further evaluate the protein expression of TRPV4 in COPD, we carried out immunohistochemistry on lung sections from COPD patients. Immunohistochemistry analysis detected increased TRPV4 protein expression in AECs from COPD patients and smokers compared to HNS (Figures 3A–C,F). Similarly, TRPV4 expression was upregulated in AECs from CS-exposed mice as compared to the air-exposed ones (Figures 3D,E,G). CS-exposed mice also showed higher *TRPV4* mRNA levels in lung tissues, as compared to the air-exposed controls (Figure 3H). Furthermore, Western blotting analysis observed increased TRPV4 protein level in lung homogenates from CS-exposed mice (Figures 3I,J). Taken together, these results demonstrated that TRPV4 was upregulated in AECs from COPD patients and long-term CS-exposed mice.

### Transient Receptor Potential Cation Channel Subfamily V Member Mediates CS Extract-Induced Pyroptosis via the Ca^2+^/NACHT, LRP, PYD Domains-Containing Protein 3/Caspase-1/Gasdermin D Axis

**FIGURE 4:**
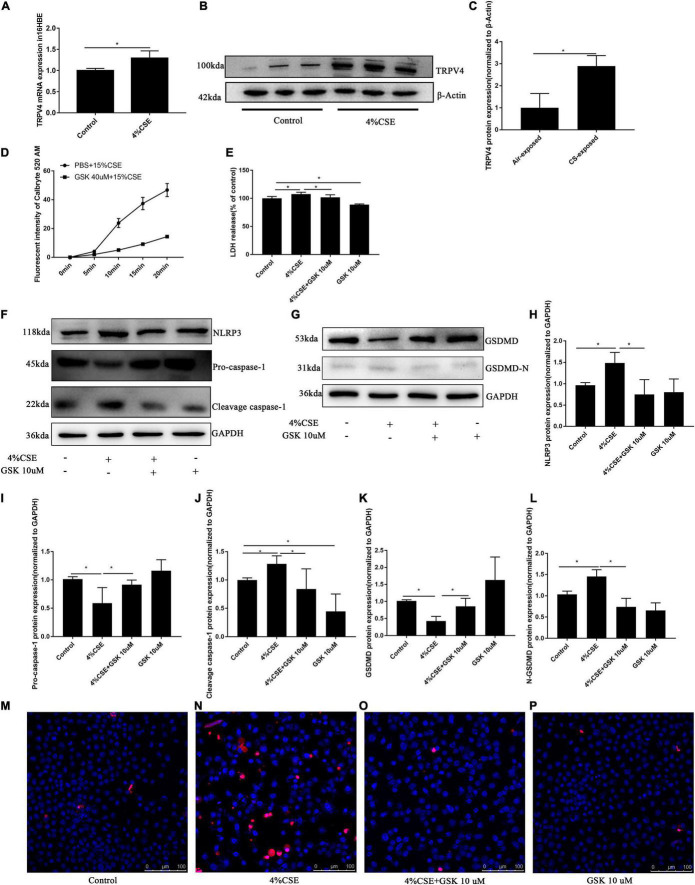
TRPV4 mediates CSE-induced pyroptosis via the Ca^2+^/NLRP3/Caspase-1/GSDMD axis. 16HBEs were pretreated with GSK205 (10 μM) for 1 h, then stimulated with 4% CSE for another 24 h. **(A–C)** CSE induced upregulated expression of *TRPV4* at mRNA level and protein level. *P < 0.05. *N* = 3 independent experiments. **(D)** Pharmacological inhibition with GSK 205 blocked Ca^2+^ influx induced by CSE. *N* = 10 cells. **(E)** LDH release induced by CSE was abrogated by pharmacological inhibition of TRPV4 with GSK 205. *P < 0.05. *N* = 6 independent experiments. **(F–L)** Pharmacological inhibition with GSK 205 decreased the level of NLRP3, Pro-Caspase-1, Cleavage cspase-1, GSDMD, GSDMD-N induced by CSE. *P < 0.05. *N* ≥ 3 independent experiments. **(M–P)** Pharmacological inhibition with GSK 205 abrogated PI positive cell induced by CSE. Bar: 100 μm.

Emerging evidence indicates that CS exposure can induce pyroptosis of AECs, suggesting a critical role of pyroptosis in the pathogenesis of COPD (Zhang et al., 2021), but the molecules mediating CS-induced pyroptosis was not clear. To determine whether TRPV4 mediates pyroptosis in CS-exposed 16HBEs, we performed pharmacological inhibition or knockdown of TRPV4 function in 16HBEs before stimulation with CSE. TRPV4 siRNA reduced TRPV4 mRNA level by 90% and protein level by 70% (Supplementary Figures 3A–C). To determine the optimal dose of CSE for *in vitro* experiments, we firstly assessed the cellular toxicity of CSE at concentrations ranging from 2 to 10%. After stimulation with 4% CSE for 24 h, about 80% cells survived (Supplementary Figure 4). Accordingly, 4% CSE exposure for 24 h was applied in following experiments. In addition, TRPV4 inhibitor showed no cellular toxicity at a range from 100 nM to 100 μM (Supplementary Figure 5). After incubation with CSE for 24 h, 16HBEs were harvested for Western blotting and qRT-PCR. We found that CSE induced significant increase in TRPV4 expression at the mRNA level and the protein level (Figures 4A–C), indicating that CS promoted TRPV4 expression, which was consistent with our observations in humans and mice.

As a previous study indicated that blocking Ca^2+^ influx could effectively inhibit pyroptosis (Wang et al., 2020), we thus measured Ca^2+^ influx in 16HBEs after corresponding treatments. We noted a time-dependent Ca^2+^ influx in 16HBEs induced by CSE, while pharmacological inhibition with GSK205 significantly attenuated this Ca^2+^ influx (Figure 4D). To further confirm this observation, we carried out *in vitro* TRPV4 siRNA experiments, which demonstrated that TRPV4 gene knockdown significantly blocked Ca^2+^ influx induced by CSE (Supplementary Figure 3D). To further explore whether TRPV4 mediates pyroptosis in CS-exposed 16HBEs, we studied the Ca^2+^/NLRP3/caspase-1/GSDMD pathway. We found that CSE induced significant NLRP3 inflammasome activation and pyroptosis in 16HBE cells, as demonstrated by elevated protein levels of NLRP3 (Figures 4F,H), Cleavage Caspase-1 (Figures 4F,J), and GSDMD-N (Figures 4G,L), and decreased levels of Pro-caspase-1 (Figures 4F,I) and total GSDMD (Figures 4G,K). Interestingly, upon using pharmacological inhibition with GSK205, we observed attenuated NLRP3 inflammasome activation and pyroptosis induced by CSE, as demonstrated by decreased protein levels of NLRP3 (Figures 4F,H), Cleavage Caspase-1 (Figures 4F,J), and GSDMD-N (Figures 4G,L), and increased levels of Pro-caspase-1 (Figures 4F,I) and total GSDMD (Figures 4G,K). These results were confirmed by *in vitro* TRPV4 siRNA experiments. CSE-exposed TRPV4 siRNA 16HBEs showed decreased protein levels of NLRP3, Cleavage Caspase-1 and GSDMD-N, but increased levels of Pro-caspase-1 and total GSDMD compared with CSE-exposed wild type 16HBEs (Supplementary Figures 3F–L). As LDH release was also a marker of pyroptosis (Wu et al., 2018), we detected LDH release in supernants of 16HBEs. Our results showed that, while CSE promoted the release of LDH, pharmacological inhibition with GSK205 or TRPV4 gene knockdown attenuated CSE-induced LDH production (Figure 4E and Supplementary Figure 3E). Moreover, we found that CSE could markedly increased PI-positive cells, which could be rescued by pharmacological inhibition with GSK205 (Figures 4M–P) or TRPV4 gene knockdown (Supplementary Figures 3M–Q). Taken together, these results indicate that TRPV4 mediates CSE-induced pyroptosis via the Ca^2+^NLRP3/Caspase1/GSDMD pathway.

### Transient Receptor Potential Cation Channel Subfamily V Member Mediates CS Extract-Induced Inflammatory Gene Upregulation and Anti-Oxidant Gene Downregulation

**FIGURE 5:**
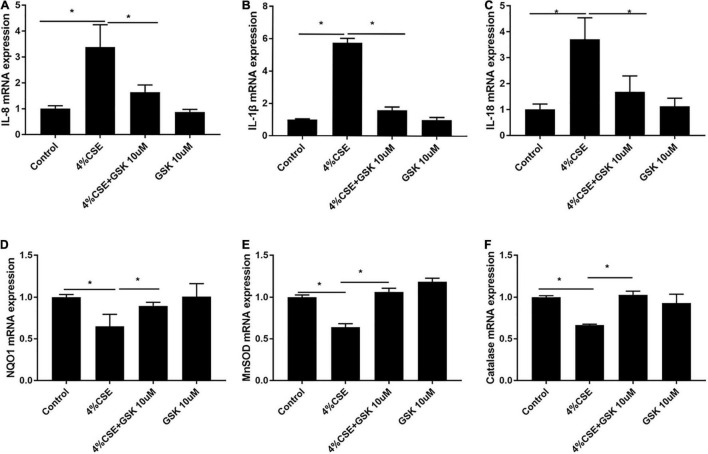
Pharmacological inhibition of TRPV4 channel with GSK205 attenuated inflammatory genes while upregulated antioxidant gene expression. 16HBEs were pretreated with GSK205 (10 μM) for 1 h, then stimulated with 4% CSE for another 24 h. **(A–C)** Increased *IL-8*, *IL-1*β, *IL-18* mRNA expression induced by CSE were abrogated by pharmacological inhibition with GSK205. **(D–F)** Decreased *NQO1*, *MNSOD*, and *Catalase* mRNA expression induced by CSE were improved by pharmacological inhibition with GSK205. *P < 0.05. *N* = 3 independent experiments.

As CS-induced injury to AECs triggers production and releasing of cytokines, which lead to recruitment of macrophages and neutrophils, participating in the development and progression of COPD (Brusselle et al., 2011; Tuder and Petrache, 2012; Barnes, 2013), we explored whether TRPV4 was possibly involved in this process. Our results showed that pharmacological inhibition of TRPV4 with GSK205 abrogated CSE-induced gene expressions of *IL-8*, *IL-1*β, and *IL-18* (Figures 5A–C) while attenuated the downregulation of anti-oxidant genes *NQO1*, *MNSOD*, and *CAT* induced by CSE (Figures 5D–F). These findings were confirmed in CSE-exposed TRPV4 siRNA 16HBEs, by showing decreased *IL-8, IL-1β, and IL-18* gene expressions but increased NQO*1*, *MNSOD*, and *CAT* gene expressions, compared with CSE-exposed wild type 16HBEs (Supplementary Figures 6A–F). Taken together, these data demonstrate that TRPV4 mediates CSE-induced inflammatory gene upregulation and anti-oxidant gene downregulation.

### Transient Receptor Potential Cation Channel Subfamily V Member 4 Mediates CS Extract-Induced Intracellular and Mitochondrial Reactive Oxygen Species

**FIGURE 6:**
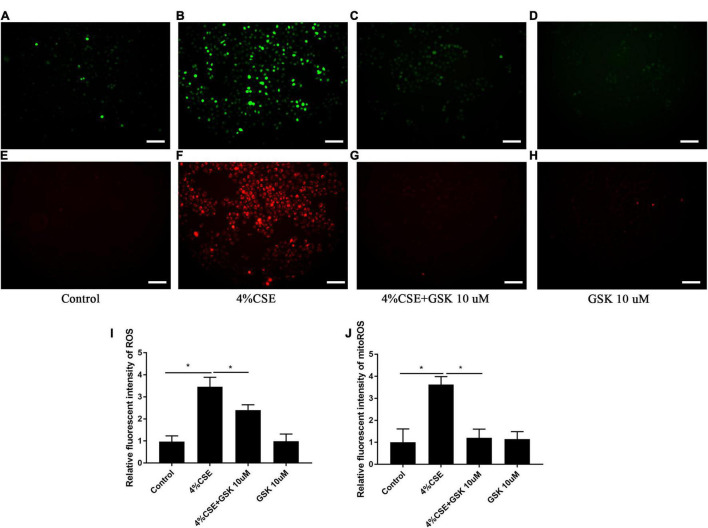
Pharmacological inhibition of TRPV4 channel with GSK205 abrogated increased intracellular and mitochondrial ROS induced by CSE. 16HBEs were treated with GSK205 (10 μM) for 1 h, then stimulated with 4% CSE for another 24 h. **(A–H)** Representative photomicrographs of intracellular and mitochondrial ROS. Increased intracellular and mitochondrial ROS induced by CSE were attenuated pharmacological inhibition with GSK205. Bar: 100 μm. **(I,J)** Quantification of photomicrographs from **(A–H)** using ImageJ software. *P < 0.05. *N* = 5 independent experiments.

As ROS and mitochondrial ROS are involved in the assembly of NLRP3 (Abderrazak et al., 2015; Zhang et al., 2021), we thus measured the intracellular and mitochondrial ROS. Exposure to CSE for 24 h significantly elevated intracellular ROS in 16HBEs, while pharmacological inhibition with GSK205 attenuated the effect of CSE (Figures 6A–D,I). Similar results were observed for the mitochondrial ROS level in these cells (Figures 6E–H,J). Additionally, this observation was further confirmed by TRPV4 siRNA experiments with 16HBEs (Supplementary Figures 7A–L).

### Transient Receptor Potential Cation Channel Subfamily V Member 4 Mediates CS Extract-Induced Damage of Mitochondrial Fitness

**FIGURE 7:**
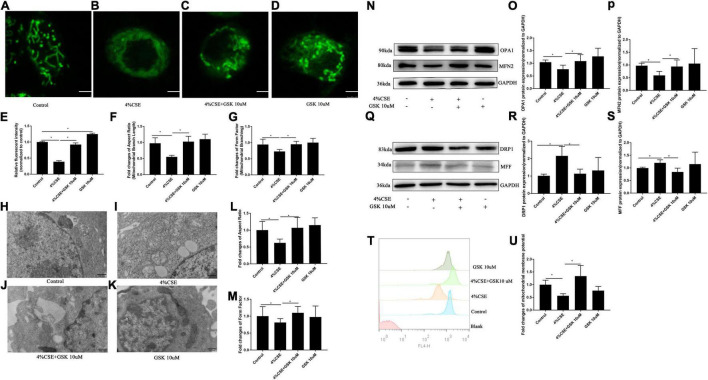
Pharmacological inhibition of TRPV4 channel with GSK205 rescued mitochondrial damage induced by CSE. 16HBEs were treated with GSK205 (10 μM) for 1 h, then stimulated with 4% CSE for another 24 h. **(A–D)** Representative images of mitochondrial morphology detected by the MitoTracker Green. Bar: 2.5 μm. **(E–G)** Quantification of Mitotracker Green intensity, Aspect Ratio and Form Factor. Decreased MitoTracke Green intensity, Aspect Ratio and Form Factor induced by CSE were rescued by pharmacological inhibition with GSK205. *P < 0.05. **(H–K)** Representative images of mitochondrial morphology detected by TEM. Bar: 500 nm. **(L,M)** Quantification of Aspect Ratio and Form Factor. Decreased Aspect Ratio and Form Factor induced by CSE were rescued by pharmacological inhibition with GSK205. *P < 0.05. **(N–P)** Pharmacological inhibition with GSK205 rescued decreased protein level of OPA1 and MFN2 induced by CSE, **(Q–S)** while mitigated increased protein level of DRP1 and MFF induced by CSE. *P < 0.05. *N* ≥ 5 independent experiments. **(T,U)** Pharmacological inhibition with GSK205 rescued decreased MMP induced by CSE. *P < 0.05. *N* ≥ 3.

Mitochondrial dysfunction is known to drive the inflammation associated with COPD (Wiegman et al., 2015). We detected the mitochondrial morphology and activity of 16HBEs after stimulation with CSE. For the detection of mitochondrial morphology, mitochondria were labeled with MitoTracker Green. The mitochondria in CSE-stimulated cells showed decreased relative fluorescence intensity, which could be rescued by pharmacological inhibition with GSK205 (Figures 7A–E) or by TRPV4 siRNA (Supplementary Figures 8A–F). Moreover, mitochondrial morphology was quantified using Aspect Ratio (length, parameter of mitochondrial fusion) and Form Factor (degree of branching, parameter of mitochondrial networking). The result demonstrated that CSE exposure also caused significant reduction of Aspect Ratio and Form Factor, which could be rescued by pharmacological inhibition with GSK205 (Figures 7F,G), or similarly by TRPV4 siRNA (Supplementary Figures 8G,H). Furthermore, ultrastructural analyses by transmission electron microscopy (TEM) observed a significant accumulation of damaged mitochondria with swollen and disrupted cristae in CSE-stimulated cells, which was mitigated by pharmacological inhibition with GSK205 (Figures 7H–K), as quantified by Aspect Ratio and Form Factor, parameters that reflect the complexity of mitochondrial morphology (Figures 7L,M). Likewise, TRPV4 siRNA 16HBEs showed alleviated mitochondrial damage compared to wild type 16HBEs (Supplementary Figures 8I–O). For the detection of mitochondrial activity, Western blotting analysis was carried out, which showed that CSE exposure for 24 h suppressed the expression of mitochondrial fusion protein OPA1 and MFN2, while pharmacological inhibition with GSK205 attenuated this suppression (Figures 7N–P). Moreover, exposure to CSE for 24 h enhanced the expression of mitochondrial fission proteins DRP1 and MFF, which could be abrogated by pharmacological inhibition with GSK205 (Figures 7Q–S). Compared to CSE-exposed wild type 16HBEs, TRPV4 siRNA 16HBEs showed increased protein levels of OPA1 and MFN2 while decreased DRP1 and MFF (Supplementary Figures 8P–U). Additionally, flow cytometry analysis showed decreased MMP after CSE stimulation, while pharmacological inhibition with GSK205 (Figures 7T,U) or TRPV4 siRNA (Supplementary Figures 8V,W) attenuated this decline. In summary, these data indicate that TRPV4 mediates CSE-induced damage of mitochondrial fitness.

## Discussion

In the present study, for the first time to our knowledge, we observed heightened pyroptosis of AECs in both human and mouse COPD. At the same time, we also confirmed upregulation of TRPV4 in these patients and the animal model. More importantly, we found that TRPV4 mediated CSE-induced pyroptosis via the Ca^2+^/NLRP3/Caspase-1/GSDMD axis, revealing a novel mechanism potentially involved in the pathogenesis of COPD.

Pyroptosis is a newly discovered form of proinflammatory cell death, defined as gasdermin-mediated programmed necrosis (Man et al., 2017; Manthiram et al., 2017; Swanson et al., 2019). Previous studies indicated that Caspase-1/4/5/11 could induce pyroptosis via cleaving gasdermin D (GSDMD) (Kayagaki et al., 2015; Shi et al., 2015). During cleavage, the inhibitory GSDMD-C domain was removed while the pore-forming GSDMD-N domain was unleashed for lysing the membranes (Aglietti et al., 2016; Ding et al., 2016; Liu et al., 2016; Sborgi et al., 2016). Although an *in vitro* study has implicated pyroptosis in the pathogenesis of COPD (Zhang et al., 2021), the expressions of pyroptosis in human and mouse COPD were not clear. In the current study, we found increased expressions of GSDMD-N/GSDMD-C in AECs from both patients with COPD and a well-established mouse model of COPD. Additionally, our *in vitro* experiments demonstrated that CSE induced upregulation of GSDMD-N in 16HBEs.

Up till now, the molecular pathways regulating pyroptosis in COPD remain to be investigated. The transient receptor potential (TRP) family is a large family of ion channel proteins which are subdivided into six groups: TRPV, TRPM, TRPA, TRPML, TRPP, and TRPC (Clapham, 2003; Negri et al., 2019). Accumulating evidence indicates that TRPV4 is involved in a variety of lung diseases, including cough (Bonvini et al., 2016; Bonvini and Belvisi, 2017), asthma (Yao et al., 2019), COPD (Baxter et al., 2014), idiopathic pulmonary fibrosis (Riteau et al., 2010), and acute respiratory distress syndrome (Balakrishna et al., 2014). However, the roles of TRPV4 in lung diseases are complex, since both protective and deleterious actions have been reported, depending on the models under study (Scheraga et al., 2020). Interestingly, a previous study indicated that TRPV4 was involved in the pathogenesis of COPD, and CSE-induced ATP and IL-1β release *in vitro* and *in vivo* (mouse model) was mediated by TRPV4 (Baxter et al., 2014), indicating that TRPV4 may be a potential therapeutic target for COPD. Emerging data supported that Ca^2+^ influx is critical for pyroptosis (Wang et al., 2020), however, the mechanisms by which Ca^2+^ influx promotes pyroptosis via the TRPV4 channel have not been elucidated. Thus, we asked whether TRPV4 mediated pyroptosis of AECs induced by CS exposure. In the current study, we demonstrated that CSE stimulation promoted Ca^2+^ influx, increased the expression of NLRP3, caspase-1, GSDMD-N, and increased the number of PI^+^ cells, which are consistent with previous reports (Wang et al., 2019; Yoo et al., 2020). Interestingly, we found that pharmacological inhibition with GSK205 or knockdown of TRPV4 function attenuated CSE-induced pyroptosis through the Ca^2+^/NLRP3/caspase-1/GSDMD pathway, as shown by decreased NLRP3, caspase-1, GSDMD-N expression, and decreased PI^+^ cell numbers, indicating that TRPV4 is critical for CS-induced pyroptosis. In addition, we observed increased IL-1β, IL18, and LDH release in 16HBEs exposed to CSE, which was in line with the consequence of enhanced pyroptosis. In fact, previous studies had reported upregulation of IL-1β and IL-18 in bronchoalveolar lavage from mouse models of COPD (Rovina et al., 2009; Sapey et al., 2009) as well as from COPD patients (Lappalainen et al., 2005; Lucattelli et al., 2011).

It is well-known that Ca^2+^ signaling is also critical for ROS and mitochondrial ROS (Jiang et al., 2011; Güzel et al., 2021), which are involved in the assembly of NLRP3 (Abderrazak et al., 2015; Zhang et al., 2021). In the current study, we observed that CSE induced ROS and mitochondrial ROS production via TRPV4 channel. Emerging evidence indicates that dysfunctional mitochondria enhanced inflammation in COPD (Wiegman et al., 2015). In our study, we observed an imbalance of mitochondrial fission and fusion proteins induced by CSE, while pharmacological inhibition with GSK205 or knockdown of TRPV4 function may be capable of preventing this abnormality, providing further evidence supporting TRPV4 as a potential candidate of therapy for CS-associated COPD.

One of the limitations of our study is that, although we demonstrated that TRPV4 mediated CSE-induced pyroptosis via the Ca^2+^/NLRP3/Caspase-1/GSDMD axis *in vitro* experiments, the significance of pyroptosis and this signaling pathway in COPD pathogenesis still needs further investigation in mouse models and human COPD.

## Conclusion

**FIGURE 8:**
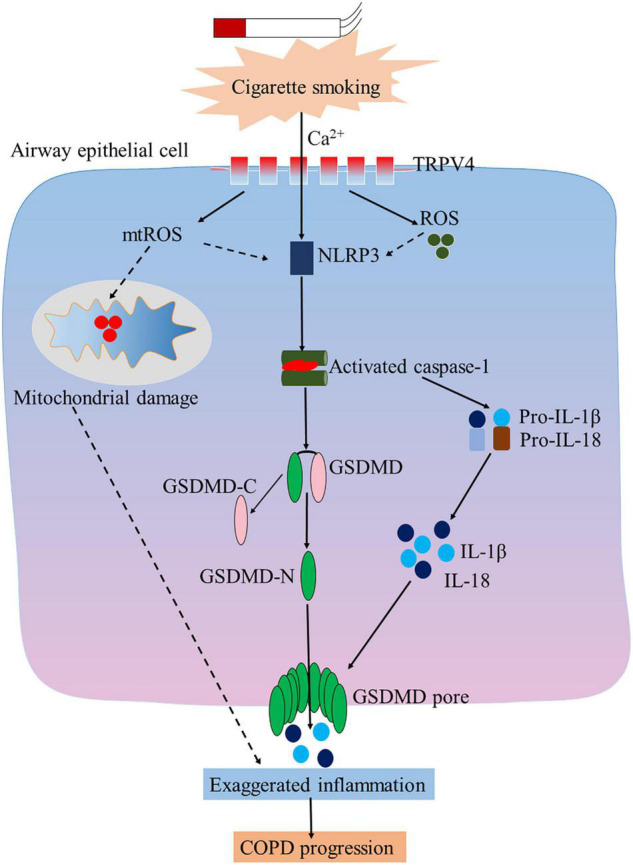
TRP4 mediates CSE-induced pyroptosis airway epithelial cells. Inhibition of TRPV4 could alleviate CSE-induced inflammation, oxidative stress, and mitochondrial damage in airway epithelial cells.

In conclusion, as shown in the proposed schematic model (Figure 8), our study reveals a novel role of TRPV4 in mediating pyroptosis of AECs from COPD. TRPV4 mediated CSE-induced pyroptosis via the Ca^2+^/ NLRP3/Caspase-1/GSDMD axis, suggesting a potential mechanism involved in the pathogenesis of COPD. TRPV4 also mediated mitochondrial damage induced by CSE. These results provide evidence for a new pathway in the pathogenic mechanisms of the disease, and hopefully for exploring interventional target for therapy of COPD.

## Data Availability Statement

The raw data supporting the conclusions of this article will be made available by the authors, without undue reservation.

## Ethics Statement

The studies involving human participants were reviewed and approved by the Ethics Review Committee of Peking University Third Hospital. The patients/participants provided their written informed consent to participate in this study. The animal study was reviewed and approved by the Animal Care Committee of Peking University Third Hospital.

## Author Contributions

YR and YS designed the experiments and wrote the manuscript. YR, XG, JX, and YL performed the experiments. All authors contributed to the article and approved the submitted version.

## Conflict of Interest

The authors declare that the research was conducted in the absence of any commercial or financial relationships that could be construed as a potential conflict of interest.

## Publisher’s Note

All claims expressed in this article are solely those of the authors and do not necessarily represent those of their affiliated organizations, or those of the publisher, the editors and the reviewers. Any product that may be evaluated in this article, or claim that may be made by its manufacturer, is not guaranteed or endorsed by the publisher.
