# Supplementary material for: Transient Receptor Potential Cation Channel Subfamily V Member 4 Mediates Pyroptosis in Chronic Obstructive Pulmonary Disease
Source: Front Physiol. 2021 Dec 24;12:783891. doi: 10.3389/fphys.2021.783891 (PMC8740047; doi:10.3389/fphys.2021.783891)
Supplement: Supplementary file 1 [file Data_Sheet_1.docx]

Supplementary Materials


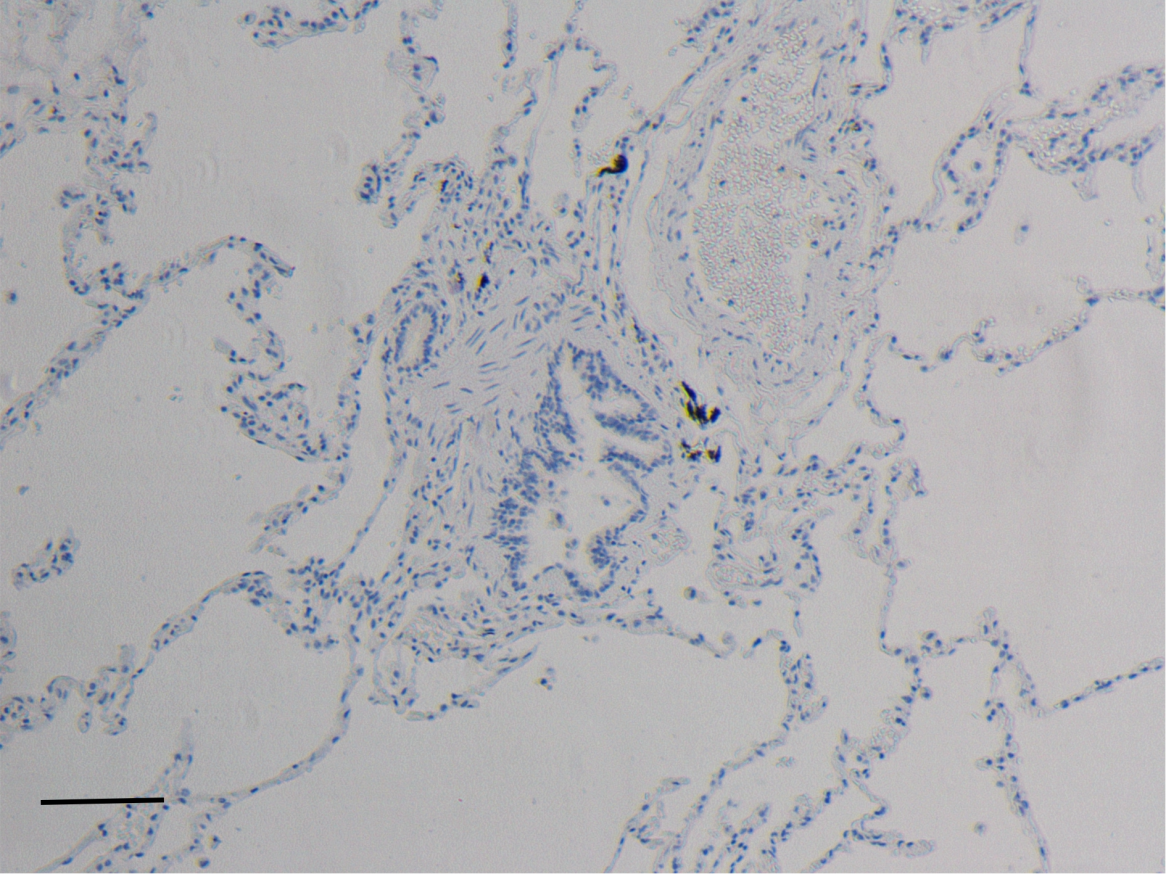


**Supplementary Figure1**: Human immunostaining control antibody staining (Bar: 100 μm.)


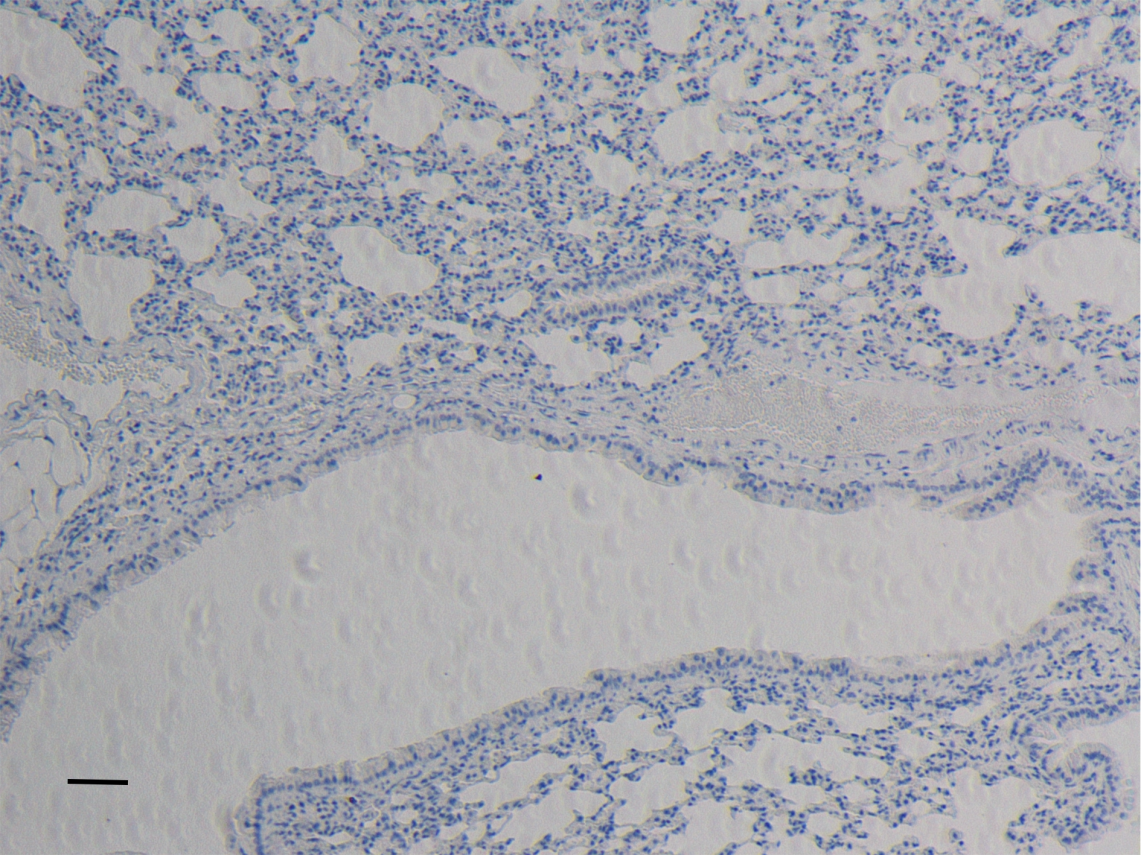


**Supplementary Figure2**: Mouse immunostaining control antibody staining (Bar: 100 μm.)


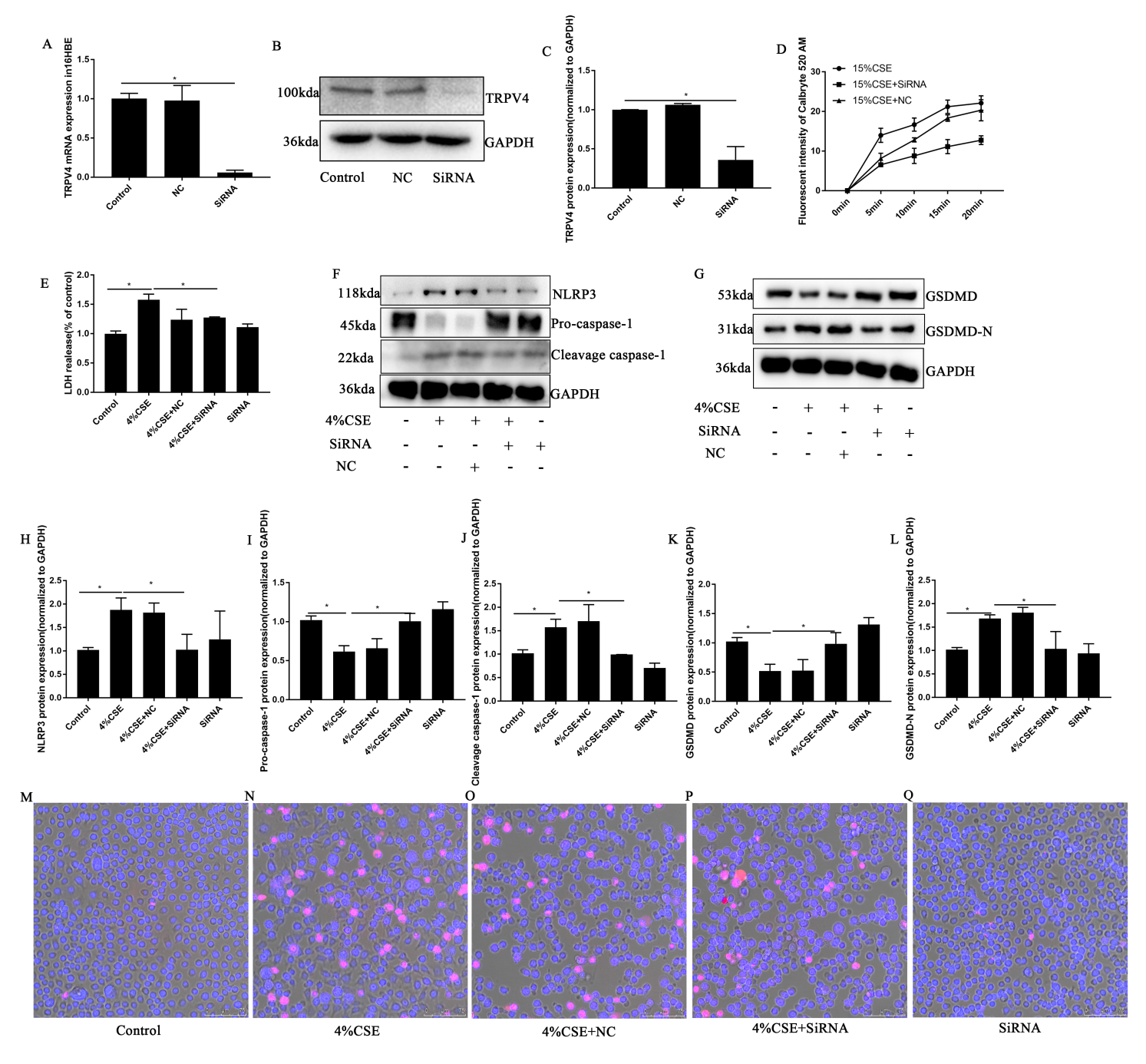


**Supplementary Figure 3. TRPV4 mediates CSE-induced pyroptosis via the Ca^2+^/NLRP3/Caspase-1/GSDMD axis.** 16HBEs were incubated with TRPV4 siRNA or scrambled siRNA for 24hrs, then stimulated with CSE for another 24h. (A)TRPV4 siRNA reduced TRPV4 mRNA level by 90% ; (B) Representative immunoblots show knockdown of TRPV4 proteins by TRPV4-specific siRNA. Quantification of (C) TRPV4/GAPDH protein bands from B. *P < 0.05.(**D**) TRPV4 gene knockdown blocked **Ca**^2+^ influx induced by CSE. N = 10 cells. (**E**) LDH release induced by CSE was abrogated inTRPV4 gene knockdown 16HBEs compared with wild type 16HBEs. * *P* < 0.05. N = 6 independent experiments. (**F-L**) TRPV4 gene knockdown in 16HBEs demonstrated decreased levels of NLRP3, Pro-Caspase-1, Cleavage cspase-1, GSDMD, GSDMD-N induced by CSE compared with wild type 16HBEs. **P* < 0.05. N ≥ 3 independent experiments. **(M-Q)** TRPV4 gene knockdown reduced PI positive cells induced by CSE. Bar: 100μm.


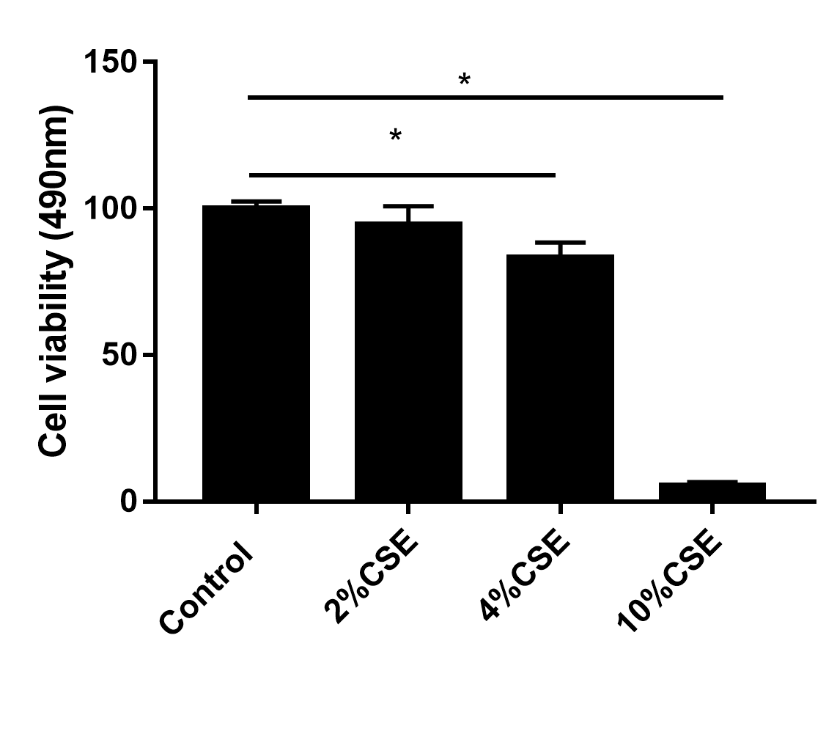


**Supplementary Figure 4.** Various concentrations of CSE were used to stimulate16HBE cells for 24 h, and then CCK8 assays were used to measure the cell viability. *P* < 0.05. N =6.

**
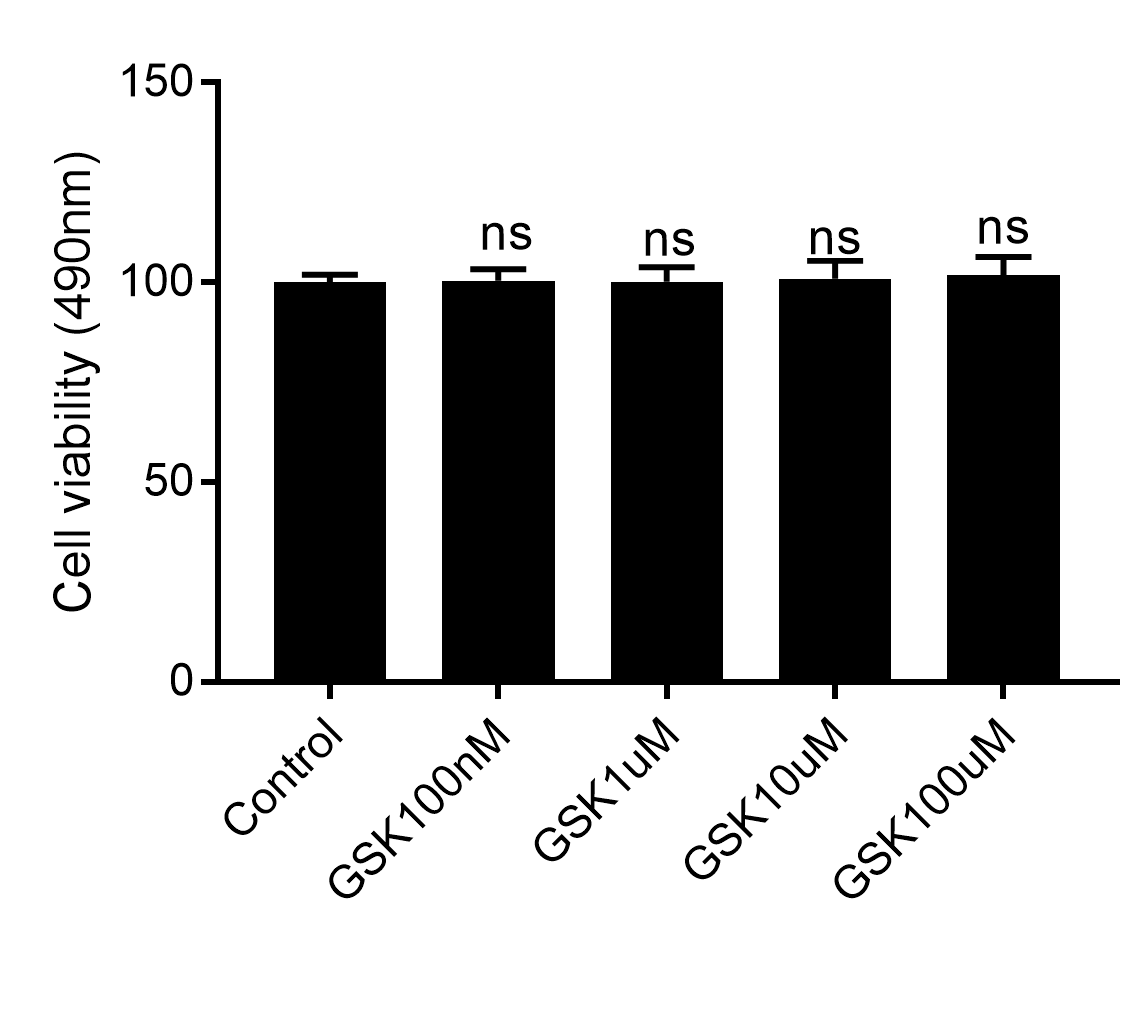
**

**Supplementary Figure 5.** Various concentrations of GSK205 were used to stimulate16HBE cells for 24 h, and then CCK8 assays were used to measure the cell viability. *P* < 0.05. N =6.


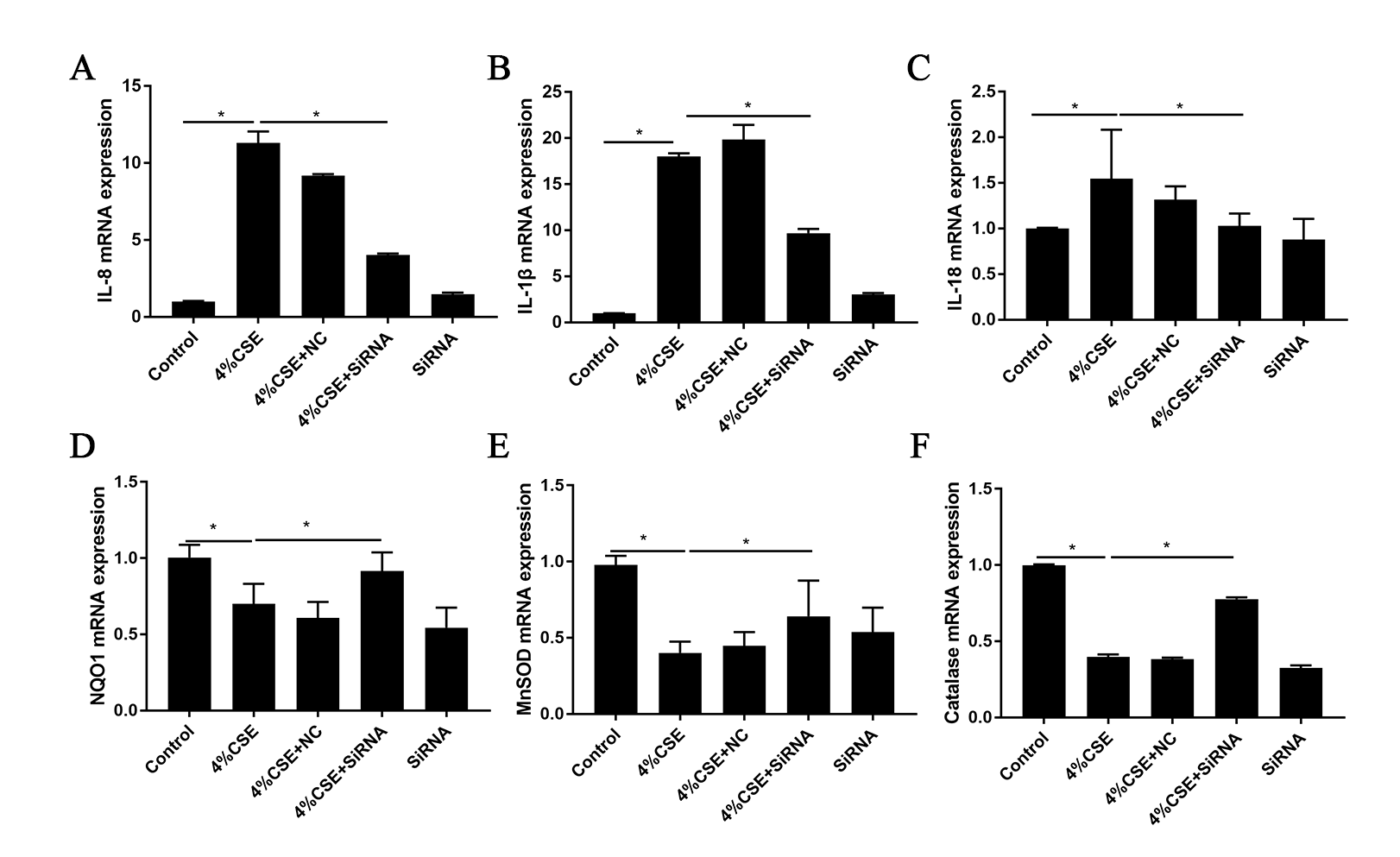


**Supplementary Figure 6. Gene knockdown of TRPV4 attenuated inflammatory genes while upregulated antioxidant gene expression.** 16HBEs were incubated with TRPV4 siRNA or scrambled siRNA for 24h, and then stimulated with CSE for another 24h.(**A-F**) Increased *IL-8*, *IL-1*β, *IL-18*, *NQO1*, *MNSOD* and *Catalase* mRNA expression induced by CSE were abrogated by gene knockdown of TRPV4. **P* < 0.05. N = 3 independent experiments.


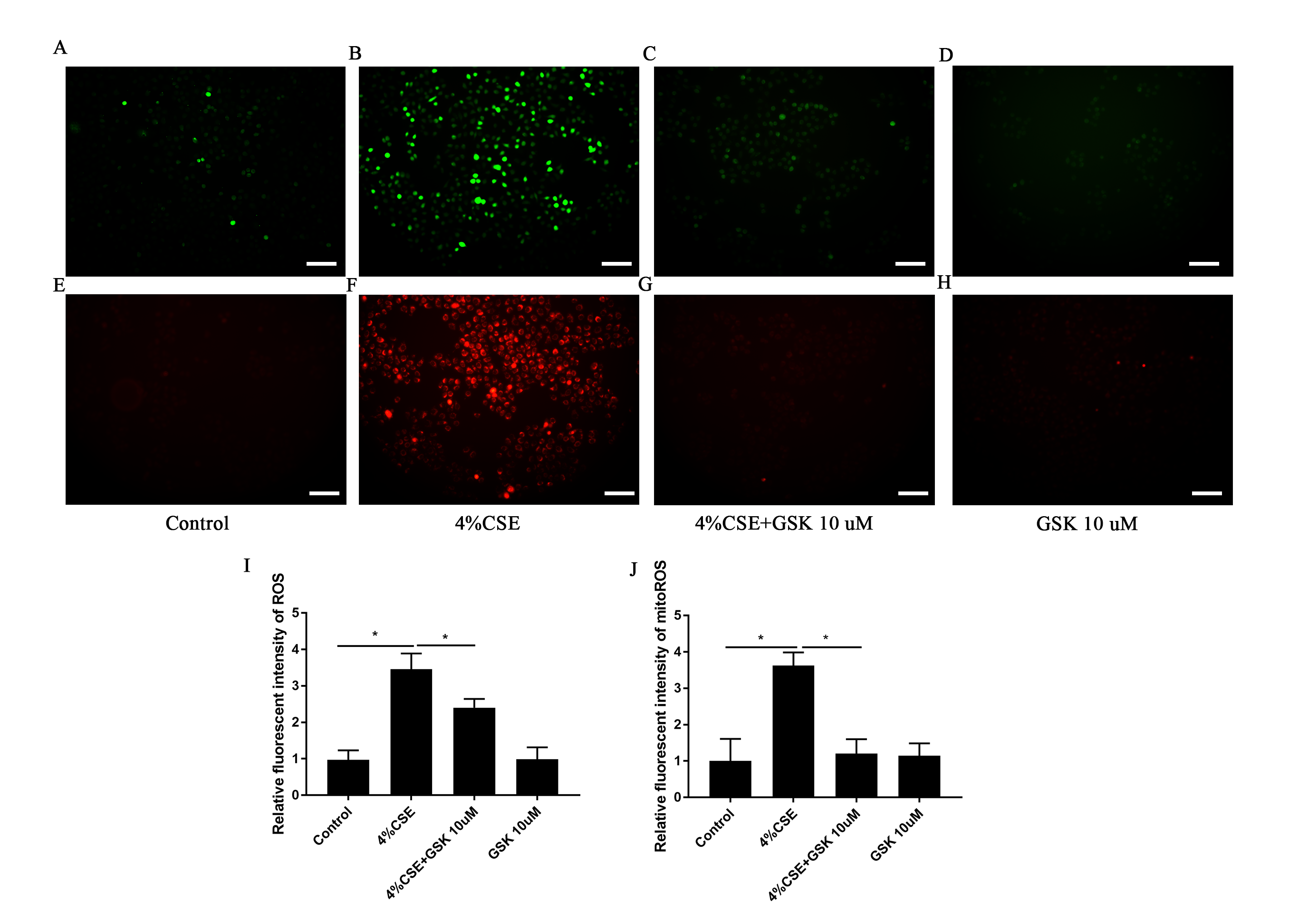


**Supplementary Figure 7. Gene knockdown of TRPV4 abrogated increased intracellular and [mitochondrial ROS](https://www.sciencedirect.com/topics/medicine-and-dentistry/mitochondrial-ros) induced by CSE.** 16HBEs were incubated with TRPV4 siRNA or scrambled siRNA for 24h, and then stimulated with CSE for another 24h. **(A-E, F-J)** Representative photomicrographs of intracellular and mitochondrial ROS. Increased intracellular and mitochondrial ROS induced by CSE were attenuated by TRPV4 knockdown. Bar: 100 μm. (**K, L**) Quantification of photomicrographs from **(A-E, F-J)** using ImageJ software. **P* < 0.05. N = 5 independent experiments.


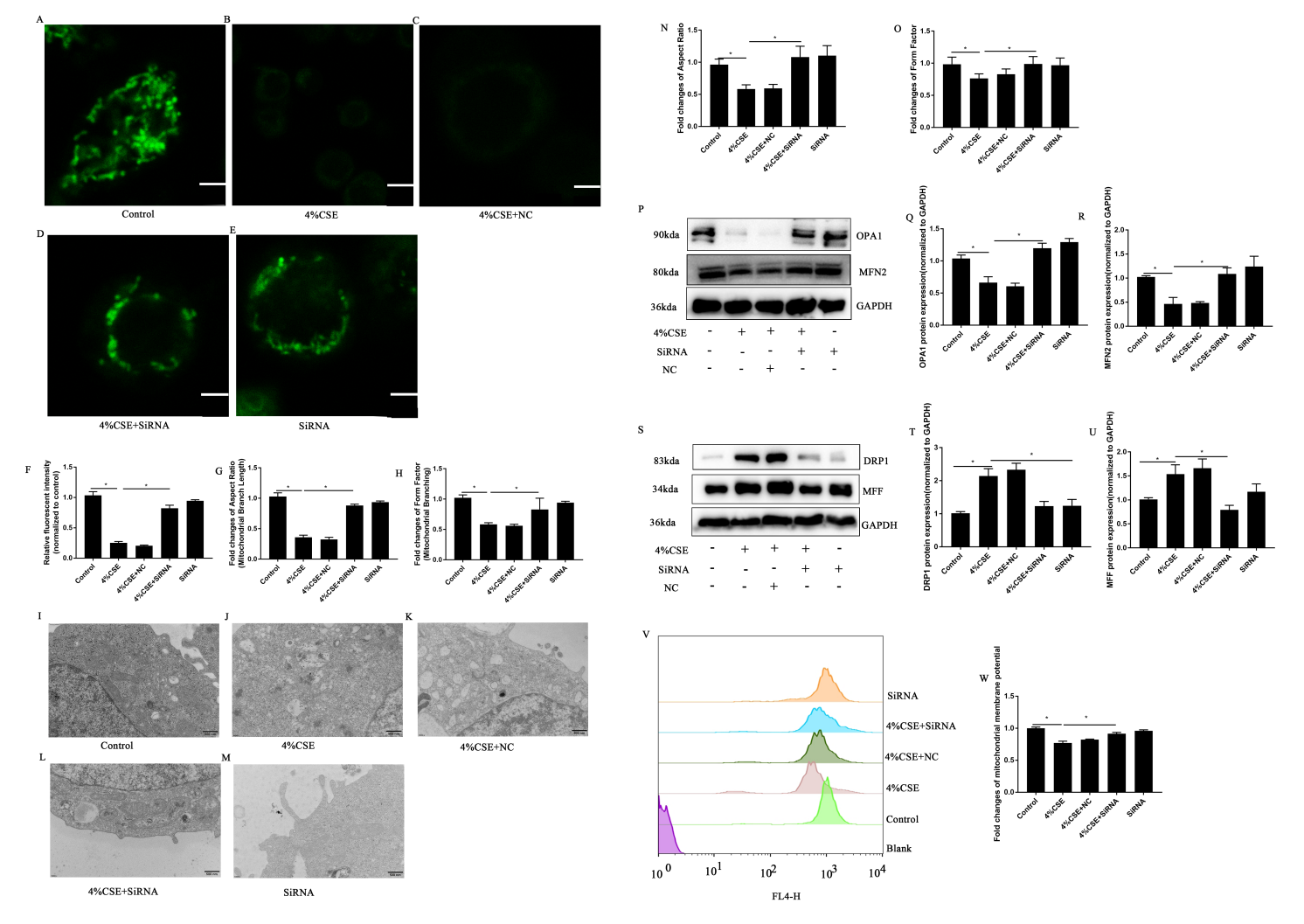


**Supplementary Figure 8. Gene knockdown of TRPV4 rescued mitochondrial damage induced by CSE.** 16HBEs were incubated with TRPV4 siRNA or scrambled siRNA for 24, and then stimulated with CSE for another 24h. (**A-E**) Representative images of mitochondrial morphology detected by the MitoTracker Green. Bar: 2.5 um. (**E-H**) Quantification of Mitotracker Green intensity, Aspect Ratio and Form Factor. Decreased MitoTracker Green intensity, Aspect Ratio and Form Factor induced by CSE were rescued by gene knockdown of TRPV4. *P < 0.05.(**I-M**) Representative images of mitochondrial morphology detecte d by TEM. Bar: 500 nm. (**N-O**) Quantification of Aspect Ratio and Form Factor. Decreased Aspect Ratio and Form Factor induced by CSE were rescued by gene knockdown of TRPV4. *P < 0.05.(**P-R**) Gene knockdown of TRPV4 rescued decreased protein level of OPA1 and MFN2 induced by CSE, **(S-U)** while mitigated increased protein level of DRP1and MFF induced by CSE. **P* < 0.05. N ≥ 3 independent experiments. **(T-U)** Gene knockdown of TRPV4 rescued decreased MMP induced by CSE. **P* < 0.05. N ≥ 3.
